# The Watcher

**Published:** 1889-03-30

**Authors:** 


### THE WATCHER.

IN hospitals the penalty imposed on a night nurse for sleeping when on duty is instant dismissal on discovery—a penalty righteous enough, since no one can say what untoward accident, even to the point of suicide, might occur with the patient, the guardian being so disabled. And in private life a person who undertakes to watch by a sick-bed and falls asleep should suffer a similar penalty, with whatever disgrace attaches. It is, however, no light task to sit in a darkened room beside a bed with a patient for whom there is little to do—a spoonful of nourishment or some drops of medicine to be administered once an hour, or a pillow beaten up, or a bit of ice dropped between the lips: to do this, and to do no more, and to keep awake. It is far easier to be on the feet all night, and have something to fill and overflow each moment with active duty, even although one be more fatigued in the morning. But, easy or difficult, it is the watcher's business to keep awake and alert, and her disgrace to sleep and be roused by the patient's voice, and if she be a paid watcher, then it becomes dishonesty. Nor must a watcher let herself be lost in the pages of an enthralling novel, which may make her oblivious to the passage of time in the scene about her; nor can she indulge herself in conversation or argument with the sick person. In fact, the watcher is to be superior to most of those weaknesses to which human beings are subject in the small hours. She has death and disease to battle with, and if once in a while she looks out of the window at the great procession of stars moving overhead, or at some whirling, howling storm without, she will feel that in the order of the universe she has mighty aids in the fight on the side of health and order, and so take heart for her work.—From "Harper's Bazaar."

